# Silencing of adaptor protein SH3BP2 reduces KIT/PDGFRA receptors expression and impairs gastrointestinal stromal tumors growth

**DOI:** 10.1002/1878-0261.12332

**Published:** 2018-06-30

**Authors:** Eva Serrano‐Candelas, Erola Ainsua‐Enrich, Arnau Navinés‐Ferrer, Paulo Rodrigues, Alfonso García‐Valverde, Sarah Bazzocco, Irati Macaya, Joaquín Arribas, César Serrano, Joan Sayós, Diego Arango, Margarita Martin

**Affiliations:** ^1^ Biochemistry Unit, Biomedicine Department Faculty of Medicine University of Barcelona Spain; ^2^ Laboratory of Clinic and Experimental Immunoallergy IDIBAPS Barcelona Spain; ^3^ Group of Biomedical Research in Digestive Tract Tumors CIBBIM‐Nanomedicine Vall d'Hebron University Hospital Research Institute (VHIR) Autonomous University of Barcelona Spain; ^4^ Preclinical Research Program Vall d'Hebron Institute of Oncology (VHIO) Barcelona Spain; ^5^ The Catalan Institute of Research and Advanced Studies (ICREA) Barcelona Spain; ^6^ CIBERONC Barcelona Spain; ^7^ Department of Biochemistry and Molecular Biology Universitat Autónoma de Barcelona Bellaterra Spain; ^8^ Vall d'Hebron University Hospital Barcelona Spain; ^9^ Immune Regulation and Immunotherapy Group CIBBIM‐Nanomedicine Vall d'Hebron University Hospital Research Institute (VHIR) Autonomous University of Barcelona Spain

**Keywords:** apoptosis, gastrointestinal stromal tumors, KIT, PDGFRA, SH3BP2

## Abstract

Gastrointestinal stromal tumors (GISTs) represent about 80% of the mesenchymal neoplasms of the gastrointestinal tract. Most GISTs contain oncogenic KIT (85%) or PDGFRA (5%) receptors. The kinase inhibitor imatinib mesylate is the preferential treatment for these tumors; however, the development of drug resistance has highlighted the need for novel therapeutic strategies. Recently, we reported that the adaptor molecule SH3 Binding Protein 2 (SH3BP2) regulates KIT expression and signaling in human mast cells. Our current study shows that SH3BP2 is expressed in primary tumors and cell lines from GIST patients and that SH3BP2 silencing leads to a downregulation of oncogenic KIT and PDGFRA expression and an increase in apoptosis in imatinib‐sensitive and imatinib‐resistant GIST cells. The microphthalmia‐associated transcription factor (MITF), involved in KIT expression in mast cells and melanocytes, is expressed in GISTs. Interestingly, MITF is reduced after SH3BP2 silencing. Importantly, reconstitution of both SH3BP2 and MITF restores cell viability. Furthermore, SH3BP2 silencing significantly reduces cell migration and tumor growth of imatinib‐sensitive and imatinib‐resistant cells *in vivo*. Altogether, SH3BP2 regulates KIT and PDGFRA expression and cell viability, indicating a role as a potential target in imatinib‐sensitive and imatinib‐resistant GISTs.

**Abbreviations**
GFP: green fluorescent protein
GIST: gastrointestinal stromal tumors
MC: mast cell
MITF: microphthalmia‐associated transcription factor
NT: nontarget
PDGFRA: platelet‐derived growth factor receptor alpha
PH: Pleckstrin homology
PLCγ: phospholipase C gamma
SH3BP2: SH3‐binding protein 2
shRNA: short hairpin RNA
TKI: tyrosine kinase inhibitor
WT: wild‐type

## 1 Introduction

Gastrointestinal stromal tumors (GIST) arise from transformed progenitor cells committed to differentiation along the interstitial cell of Cajal lineage and are the most common mesenchymal tumors of the gastrointestinal tract (Fletcher *et al*., 2002). GISTs are characterized by activating mutations in *KIT* and *PDGFRA* genes which are shown to be mutually exclusive (Gasparotto *et al*., 2008). KIT is structurally similar to platelet‐derived growth factor receptor alpha (PDGFRA) and belongs to the type III receptor tyrosine kinase family (Rosnet *et al*., 1991; Yarden *et al*., 1987).

Between 65% and 85% of GISTs have KIT mutations (Heinrich *et al*., 2003a; Miettinen *et al*., 2005; Rubin *et al*., 2001), while PDGFRA mutations are found in 5–10%. Approximately 10–15% of GISTs do not have detectable mutations in either of these receptors, suggesting that other molecules and pathways may be involved in the pathology (Lasota and Miettinen, 2008; Reichardt *et al*., 2009; Tornillo, 2014). Most KIT mutations affect the juxtamembrane domain (exon 11) involved in KIT's autoregulatory function and promote spontaneous kinase activation (Chan *et al*., 2003). Exon 9 (extracellular domain) mutations are the next most common mutations, followed by exon 13 (tyrosine kinase 1 domain) and exon 17 (tyrosine kinase 2 domain) mutations. Interestingly, PDGFRA gain‐of‐function mutations are found in around half of the GISTs lacking KIT mutations (Heinrich *et al*., 2003a; Hirota *et al*., 2003). The majority of PDGFRA mutations affect the tyrosine kinase 2 domain (exon 18). These mutations change the activation loop, which regulates the ATP‐binding pocket and leads to kinase activation (Rubin *et al*., 2007).

Imatinib mesylate, a small molecule tyrosine kinase inhibitor (TKI), is approved for first‐line treatment of metastatic and unresectable GISTs. It works by binding to the ATP‐binding sites of KIT, PDGFRA, and BCR‐ABL fusion product, consequently inhibiting their activity (Lyseng‐Williamson and Jarvis, 2001). During imatinib treatment, resistance often develops as a result of secondary mutations, primarily in the kinase domains of KIT or PDGFRA (Chen *et al*., 2004; McLean *et al*., 2005; Tamborini *et al*., 2004). Sunitinib malate, an oral multitargeted inhibitor of KIT, PDGFRA, and vascular endothelial growth factor receptor (VEGFR), is approved for the treatment of imatinib‐resistant GISTs. Regorafenib is used as a third‐line agent (Serrano *et al*., 2017). However, despite an initial response to these targeted agents, tumors invariably become resistant (Kang *et al*., 2013) and new therapeutic approaches are needed to improve the clinical management of GIST patients.

We have recently shown that the adaptor molecule SH3‐binding protein 2 (SH3BP2) regulates the expression and signaling of KIT receptor in human mast cells. SH3BP2 is a cytoplasmic adaptor originally identified as a protein interacting with the SH3 domain of the protein kinase ABL (Ren *et al*., 1993). Human SH3BP2 is a 561‐aa protein containing an N‐terminal pleckstrin homology (PH) domain, an SH3‐binding proline‐rich region, and a C‐terminal SH2 domain; it is preferentially expressed in hematopoietic tissues and has a regulatory role in adaptive and innate immune responses (Ainsua‐Enrich *et al*., 2012; Deckert *et al*., 1998; Foucault *et al*., 2005; Jevremovic *et al*., 2001).

Silencing of SH3BP2 downregulates KIT expression at transcriptional level, regulating microphthalmia‐associated transcription factor (MITF) at post‐transcriptional level and leading to an increase of apoptosis in human mast cells. Moreover, SH3BP2 regulates wild‐type KIT and mutated D816V KIT expression (Ainsua‐Enrich *et al*., 2015), a hallmark mutation in mastocytosis, which is a rare disease comprising MC leukemia characterized by aberrant growth and accumulation of clonal MCs in different organs (Metcalfe and Mekori, 2017).

Here, we present the first report of the expression of SH3BP2 in primary tumors and cell lines from GIST patients. We identify its role in cell viability and tumor growth of imatinib‐sensitive and imatinib‐resistant GIST cells *in vitro* and *in vivo*, and provide some insights into its mechanism of action.

## 2 Materials and methods

### 2.1 Antibodies and reagents

Mouse anti‐SH3BP2 (clone C5 for western blot and C11 for immunofluorescence), mouse anti‐KIT (clone Ab81) for western blot, mouse anti‐PLCγ, rabbit anti‐KIT (clone H300) for immunofluorescence, and rabbit anti‐PDGFRA (clone C20) for immunofluorescence were purchased from Santa Cruz Biotechnology, Inc. (Santa Cruz, CA, USA). Rabbit anti‐pPLCγ (Y783), anti‐AKT, anti‐pAKT 1/2/3S473 (clon193H12), anti‐pKIT(Y703) (clone D12E12), anti‐PDGFRA (polyclonal) for western blot, and anti‐MITF (clone D5G7V) were obtained from Cell Signaling Technology, Inc. (Danvers, MA, USA). Mouse anti‐α‐tubulin (clone DM1A) and anti‐β‐actin (clone AC‐40) were purchased from Sigma (St. Louis, MO, USA). Mouse antiphosphotyrosine (pTyr; clone PY20) was obtained from Zymed Laboratories (Invitrogen Life Technologies, Carlsbad, CA, USA). Anti‐mouse and anti‐rabbit IgG peroxidase Abs were purchased from DAKO (Carpinteria, CA, USA) and Bio‐Rad (Hercules, CA, USA), respectively. Imatinib and bortezomib were purchased from Sigma.

### 2.2 Cell culture

Human GIST cell lines GIST882, GIST48, and GIST48B were kindly provided by S. Bauer. GIST882 (Tuveson *et al*., 2001) were maintained in RPMI 1640 (Lonza, Basel, Switzerland) supplemented with 15% fetal bovine serum (FBS) (Attendbio; Barcelona, Spain), 1% l‐glutamine (Lonza), 50 units·mL^−1^ penicillin, and streptomycin (Lonza). GIST48 (Bauer *et al*., 2006) and GIST48B (Muhlenberg *et al*., 2009) were maintained in Ham's F‐10 (Lonza) supplemented with 15% FBS, 1% l‐glutamine, 50 units·mL^−1^ penicillin and streptomycin, 30 mg·mL^−1^ bovine pituitary extract, and 0,5% MITO+ Serum Extender (Fischer Scientific, Pittsburg, PA). GIST430/654 (Bauer *et al*., 2006) were cultured in IMDM supplemented with 15% FBS, 1% L‐glutamine, 50 units·mL^−1^ penicillin and streptomycin, and additional 200 nM imatinib mesylate (Sigma‐Aldrich) to maintain selective pressure. Imatinib‐sensitive GIST‐T1 and imatinib‐resistant GIST‐T1‐derived sublines GIST‐T1/670 and GIST‐T1/816 have been published previously (Garner *et al*., 2014). Mycoplasma test is performed routinely in all cell lines used.

### 2.3 Immunoprecipitation and immunoblotting

Immunoprecipitation and immunoblottings with the indicated antibodies were carried out as previously described (Alvarez‐Errico *et al*., 2011). Frozen tumor samples were diced in ice‐cold lysis buffer (1% NP‐40, 50 mm Tris/HCl, pH 8.0, 100 mm sodium fluoride, 30 mm sodium pyrophosphate, 2 mm sodium molybdate, 5 mm EDTA, 2 mm sodium orthovanadate) on dry ice and homogenized on ice with a Tissue Tearor Homogenizer. Cell lysates were then rocked overnight at 4 °C. Lysates were cleared by centrifugation at 12 000 rpm for 30 min at 4 °C, and lysate protein concentrations were determined using a Bio‐Rad protein assay. Electrophoresis and protein blotting were performed using standard techniques. Hybridization signals were detected by chemiluminescence (Immobilon Western, Millipore Corporation, Billerica, MA, USA) and captured using a G:BOX chemiluminescence imaging system (Syngene, Cambridge, UK).

### 2.4 Immunofluorescence

Immunofluorescence was performed as described elsewhere (Ainsua‐Enrich *et al*., 2012). Briefly, cells were seeded in poly‐lysine coverslips for each point 1 day before the experiment in complete media. After fixation with PFA 4%, cells were then permeabilized with saponin 0.05%, washed, and blocked before incubation with specific antibodies. Cells were incubated for 5 min with Hoechst 33258 (Bio‐Rad) and mounted in Fluoromount Gold (Life Technologies). Cells were visualized with a Leica TCS SP5 confocal microscope.

### 2.5 Lentiviral transduction

Lentiviral particles to silence the SH3BP2 gene expression were generated using Mission^®^ shRNA technology according to manufacturer's instructions (Sigma). Nontarget sequence was as follows: 5′CCGGCAACAAGATGAAGAGCACCAACTCGAGTTGGTGCTCTTCATCTTGTTGTTTTT 3′. The SH3BP2‐specific shRNA sequences were as follows: sequence 21:5′CCGGGCGAAGTGGAAAGGTTGTTCACTCGAGTGAACAACCTTTCCACTTCGCTTTTTG3′; sequence 17:5′CCGGGCACCCAATTATGCAAGCATTCTCGAGAATGCTTGCATAATTGGGTGCTTTTTG3′. SH3BP2‐GFP and MITF‐GFP were from OriGene Technologies, Rockville, MD. Lentiviral transduction for NT (nontarget), SH3BP2 silencing and GFP, SH3BP2‐GFP, or MITF‐GFP overexpression was performed as described in Ainsua‐Enrich *et al*. (2012, 2015) with slight modifications. Virus preparations were done by precipitating viruses with polyethylene glycol (PEG) as described in Marino *et al*. (2003). GIST cells were transduced in the presence of 8 μg·mL^−1^ of polybrene (Santa Cruz), and puromycin selection was carried out after 1 day from transduction.

### 2.6 RNA extraction, retrotranscription, and real‐time PCR

One microgram of total RNA was used for cDNA retrotranscription and posterior real‐time PCR for *KIT*,* PDGFRA,* and *MITF* expression analysis, following the protocol described elsewhere (Ainsua‐Enrich *et al*., 2012).

### 2.7 Cell viability and detection of caspase 3 and caspase 7 activities

Cell viability and caspase activity were assayed using the CellTiter‐Glo^®^ Luminescent Cell Viability Assay and Caspase‐Glo™ 3/7 Assay (Promega, San Luis Obispo, CA, USA), respectively, according to the manufacturer's instructions.

### 2.8 Wound‐healing assay

Cells were cultured in 6‐well plates and allowed to grow to 90% confluence. The cell monolayer was scratched with a sterile micropipette tip, and the wound region was allowed to heal by cell migration. The area that remained clear of cells after 0, 12, and 24 hours was quantified with ImageJ (National Institutes of Health, NIH) and compared with the area of the wound at time zero. Experiments were run three times in triplicate.

### 2.9 *In vivo* xenografts

For GIST882 xenograft experiments, seven‐week‐old female athymic nude‐foxn1 mice (Envigo; Huntingdon, UK) were injected subcutaneously in both flanks with 10^7^ GIST882 cells in complete medium. GIST430/654 xenografts were prepared by subcutaneously injecting 5 x 10^6^ cells in 50 μL serum‐free medium mixed with 50 μL Matrigel (~10 mg·mL^−1^, BD Biosciences) into both flanks of six‐ to eleven‐week‐old female BALB/c severe combined immunodeficient (SCID) mice (Envigo; Huntingdon, UK). In both cases, NT shRNA control cells were injected in the left flank and SH3BP2 shRNA cells were injected in the right flank. Tumor volume (*V*) was calculated with the formula: *V* = (L × W^2^) × 0.52, where *L* is the length and *W* is the width of the tumor.

### 2.10 Statistical data analysis

All results are expressed as mean ± standard error of the mean (SEM). After determination of normal distribution of the samples and variance analysis, unpaired Student's *t*‐test was used to determine significant differences (*P* value) between two experimental groups and one‐way ANOVA test was used to determine significant differences (*P* value) between several experimental groups.

### 2.11 Study approval

Animal protocol procedure was approved by Vall d'Hebron Ethical Committee for Animal Experimentation and for CEA‐Generalitat de Catalunya (Catalonian Government Ethical Committee) (protocol 5769). The procedure meets local and national legislation, which is a transposition of the 2010 63 EU directive. Mice were maintained at the Vall d'Hebrón animal facility in accordance with Institutional guidelines. The samples used in the current study were provided by Tumor Bank of the Vall d'Hebron University Hospital Biobank with appropriate ethical approval. This study was approved by the Institutional Review Board from Vall d'Hebron University Hospital. Informed consent was obtained from all patients prior to study enrollment.

## 3 Results

### 3.1 SH3BP2 is expressed in primary tumors from GIST patients

Recently, we showed that SH3BP2 regulates KITD816V, a gain‐of‐function mutation receptor associated with mastocytosis (Ainsua‐Enrich *et al*., 2015). Microphthalmia‐associated transcription factor (MITF), involved in KIT expression and signaling (Lee *et al*., 2011; Tsujimura *et al*., 1996), has been proposed as the target of SH3BP2 mechanism of action in mast cells (Ainsua‐Enrich *et al*., 2015). MITF has been described as an oncogene in melanoma (Garraway *et al*., 2005), but it has not been previously studied or reported in GISTs. Active KIT mutations other than KITD816V are generally found in gastrointestinal stromal tumors (GISTs), and so the purpose of this study was to analyze the role of SH3BP2 in other KIT oncogenic mutations associated with pathology.

**Figure 1:**
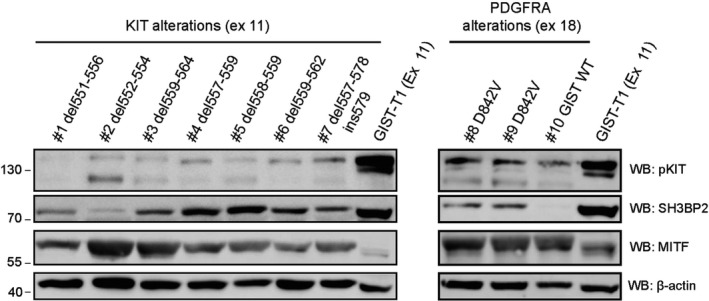
SH3BP2 and MITF are expressed in primary tumors from GIST patients. Whole cell lysates from primary tumors with different genomic alterations as indicated in the figure and GIST T1 cell line (as control) were analyzed for pKIT, SH3BP2, and MITF expression. β‐actin was used as loading control.

We first assessed the expression of SH3BP2 and MITF in primary tumors of GIST patients with a clinically representative mutation spectrum of the disease: *KIT* mutations in exon 11 and *PDGFRA* mutations in exon 18, as well as KIT/PDGFRA wild‐type (WT). The presence of KIT mutations in exons 9, 11, 13, and 17, and PDGFRA mutations in exons 12 and 18 were assessed in FFPE samples from LTR cases as previously reported (Heinrich *et al*., 2003b; Kinoshita *et al*., 2004). As can be seen in Figure 1, SH3BP2 was present in variable amounts in all tumors with KIT or PDGFRA mutations. Interestingly, it was not detected in the GIST WT. In contrast, MITF was detected in all tumors tested.

To study SH3BP2 function, we characterized three different cell lines derived from GIST patients: GIST882 (*KIT* exon 13 K642E), an imatinib‐sensitive cell line; GIST48 (*KIT* exon 11 D820A plus *KIT* exon 17 V560D), an imatinib‐resistant cell line; and GIST48B, a subline of GIST48, which, despite retaining the activating KIT mutation, expresses KIT transcript and protein at essentially undetectable levels (Muhlenberg *et al*., 2009). All GIST cell lines tested express SH3BP2 independently of their sensitivity to imatinib. Due to the gain‐of‐function mutation of KIT, we found the receptor to be phosphorylated. PDGFRA was found to be phosphorylated in GIST882 and GIST48B cells, suggesting activation of this receptor in basal conditions (Fig. S1). Interestingly, SH3BP2 showed a partial colocalization with KIT and PDGFRA (Fig. S2), suggesting that it may form part of the same signalosome complex.

### 3.2 SH3BP2 silencing correlates with KIT and PDGFRA expression and GIST cell viability

**Figure 2:**
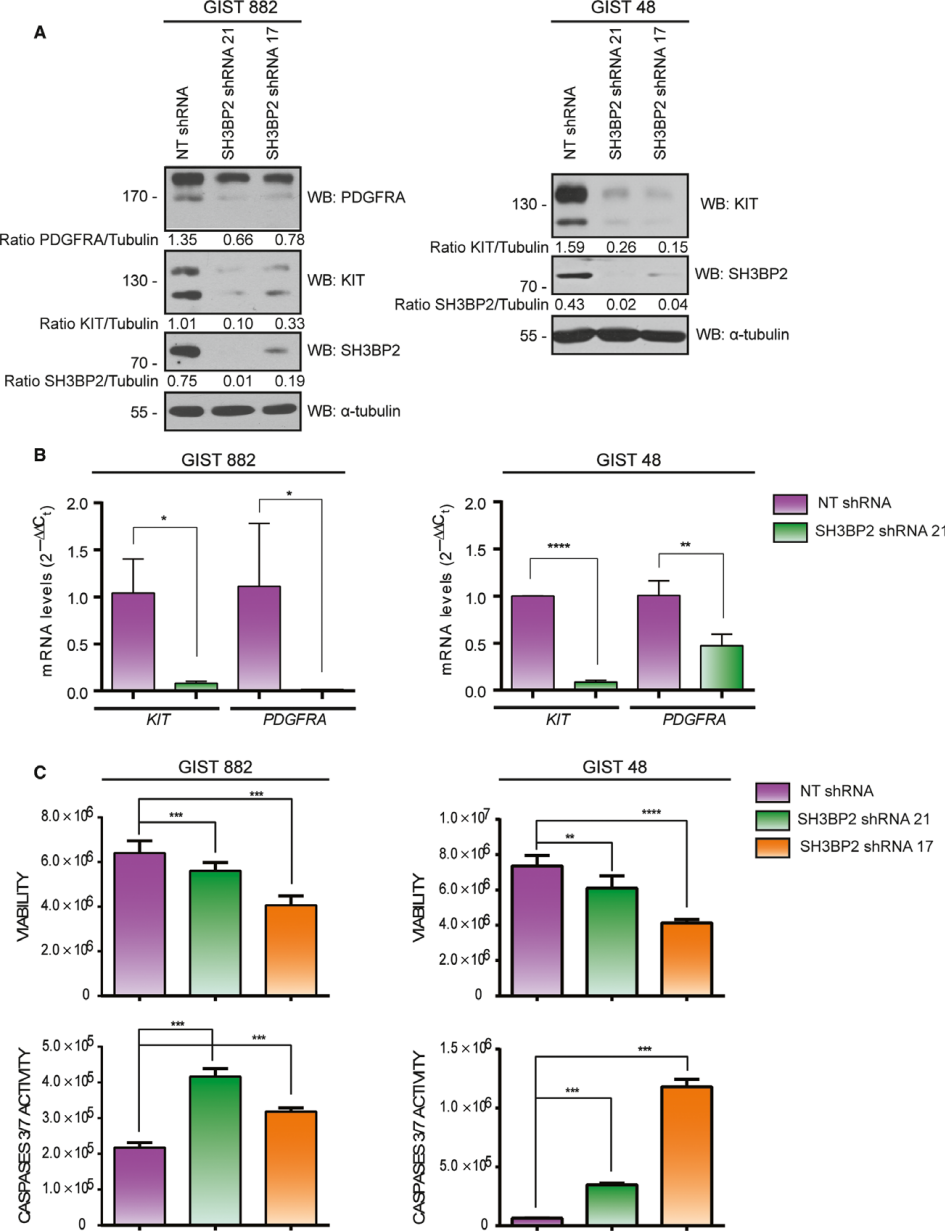
SH3BP2 silencing causes a decrease in KIT and PDGFRA expression and cell viability. GIST882 and GIST48 cells were transduced with either control NT (Nontarget) shRNA or two different sequences for SH3BP2 shRNA. (A) Lysates from transduced cells were analyzed for SH3BP2, KIT, and PDGFRA protein levels. Tubulin was used as loading control. (B) Real‐time PCR was performed from total mRNA using specific probes against *KIT* and *PDGFRA*. Statistical significance (*P <* *0.05, **P <* *0.01, ****P <* *0.0001; unpaired *t*‐test; *n *=* *3; mean ± SEM) is relative to NT shRNA. (C) Viability and caspase 3/7 activity analysis was performed in NT and SH3BP2‐silenced cells. Statistical significance (**P <* *0.01, ***P <* *0.001, ****P <* *0.0001; one‐way ANOVA with Bonferroni's post hoc test; *n *=* *3) is relative to NT shRNA.

To investigate the role of SH3BP2 in GISTs, SH3BP2 gene expression was stably silenced by lentivirus‐mediated shRNA in GIST882 and GIST48 cell lines. SH3BP2 knockdown was effective at this timing with two different shRNA (shRNA‐21 and shRNA‐17; Fig. 2A). SH3BP2 silencing was accompanied by a significant reduction in KIT expression at the protein (Fig. 2A) and mRNA levels (Fig. 2B) in both GIST cell lines. Interestingly, although PDGFRA is not expressed at protein levels in GIST48, it can be detected at mRNA levels. Notably, it was also reduced after SH3BP2 silencing in GIST48 cells (Figure 2A). These results indicate that SH3BP2 may act as a common regulator for the expression of KIT and PDGFRA receptors at transcriptional level.

We next investigated the effect of SH3BP2 silencing on cell survival. We found that the SH3BP2 knockdown reduces cell viability and increases caspase 3/7 activity (Fig. 2C), in agreement with the reduction of the expression of KIT and/or PDGFRA oncogenic receptors.

We further analyzed the effect of SH3BP2 silencing in KIT/PDGFRA‐dependent signaling, evaluating the phosphorylation of PLC gamma (PLCγ) and AKT that have been shown to be downstream of KIT/PDGFRA in GISTs (Bauer *et al*., 2007; Duensing *et al*., 2004). In GIST 882 and 48, we found a reduction in PLCγ phosphorylation, consistent with the positive role of this adaptor in PLCγ activation in mast cells reported previously by our group (Ainsua‐Enrich *et al*., 2012). AKT phosphorylation, a surrogate marker of PI3K, was also reduced consistent with the reduction in cell viability (Fig. S3).

### 3.3 The microphthalmia‐associated transcription factor is reduced after SH3BP2 silencing in GIST cells

**Figure 3:**
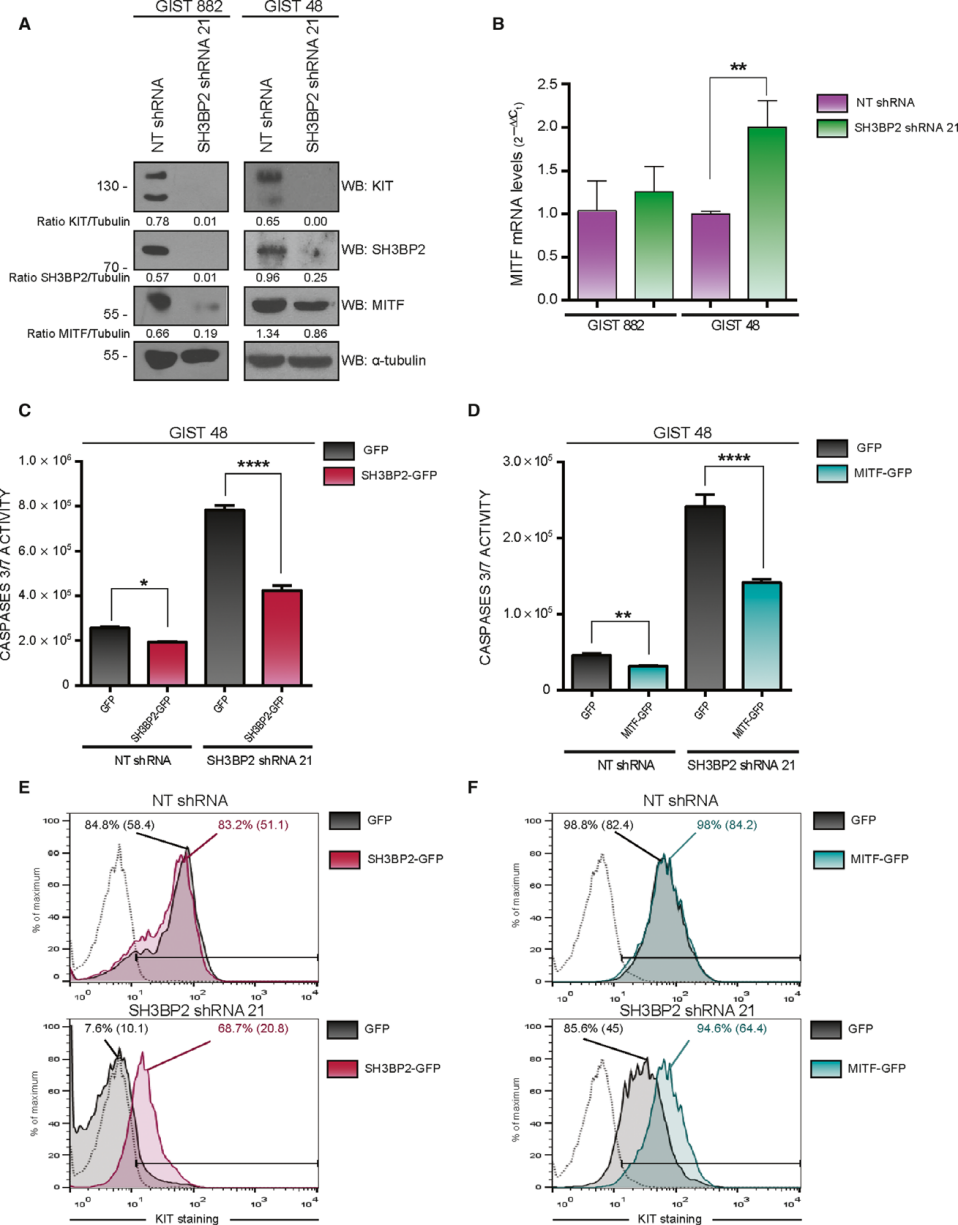
Microphthalmia‐associated transcription factor expression is reduced in SH3BP2‐silenced cells. SH3BP2 or MITF reconstitution restores cell survival. (A) GIST882 and GIST48 cells were transduced with either control NT (Nontarget) shRNA or SH3BP2 shRNA. Cell lysates were analyzed for MITF protein levels. Tubulin was used as loading control. (B) Real‐time PCR was performed using specific probes against MITF. Statistical significance (**P <* *0.01; unpaired *t*‐test; *n *=* *3; mean ± SEM) is relative to NT shRNA. (C–F) GIST48 cells transduced with either control NT (nontarget) shRNA or SH3BP2 shRNA were posteriorly reconstituted with GFP, SH3BP2‐GFP, or MITF‐GFP. Evaluation of caspase 3/7 activity was performed for SH3BP2 reconstitution (C) or MITF reconstitution (D). Statistical significance (*P <* *0.05, ****P <* *0.0001; unpaired *t*‐test; n = 3; mean ±SEM) is relative to GFP‐transduced cells in each case. KIT surface expression by flow cytometry was assayed in SH3BP2‐reconstituted (E) and MITF‐reconstituted (F) SH3BP2‐silenced cells. Percentage of KIT expression and mean fluorescence, in parentheses, are indicated in the FACS histograms.

As previously reported, SH3BP2 silencing downregulates the basic helix‐loop‐helix transcription factor microphthalmia‐associated transcription factor (MITF) expression in mast cells (Ainsua‐Enrich *et al*., 2015). MITF binds to a CACCTG motif in the *KIT* promoter and has been shown to regulate *KIT* expression in mast cells (Tsujimura *et al*., 1996). The role of MITF in GIST remains elusive so next we assessed whether MITF was involved in SH3BP2 modulation of KIT expression and cell survival in GIST. As shown in Fig. 3A, the MITF protein is reduced after SH3BP2 silencing, while MITF mRNA was not altered (Fig. 3B), indicating a post‐transcriptional mechanism in the SH3BP2‐mediated regulation of MITF expression.

### 3.4 SH3BP2 and MITF are directly involved in cell viability in GIST cells

To analyze the specificity of the effect of SH3BP2 on cell survival, SH3BP2‐silenced GIST48 cells were reconstituted with GFP‐tagged SH3BP2, or with GFP as a control. For this purpose, after two days of SH3BP2 shRNA or NT transduction, when cells were still viable, they were transduced with SH3BP2‐GFP or GFP lentivirus. Six days after the last transduction and caspase activity was measured. Our data show that SH3BP2 reconstitution significantly reverses the apoptotic phenotype (Fig. 3C). The same approach was applied to assess the effect of MITF on SH3BP2‐silenced cells, by reconstitution with MITF‐GFP following the above procedure. Our results show that overexpression of MITF (Fig. 3D) significantly reverses the phenotype produced by SH3BP2 silencing, suggesting the involvement of this transcription factor in the regulatory mechanism in which SH3BP2 expression is critical. Efficiency and specificity of reconstitution were analyzed in all cases (Fig. S4).

### 3.5 Imatinib and bortezomib alter KIT, SH3BP2, and MITF expression

Next, we tested the effect of KIT activity inhibition as well as KIT expression inhibition, on SH3BP2 and MITF expression. For this purpose, we incubated GIST882 cells with imatinib and bortezomib. Incubation of GIST882 with these drugs leads to a reduction in KIT, SH3BP2, and MITF protein levels (Fig. S5), suggesting that these proteins are commonly affected after KIT expression and activity inhibition.

### 3.6 SH3BP2 silencing also promotes PDGFRA downregulation and cell viability impairment in the GIST48B cell line

**Figure 4:**
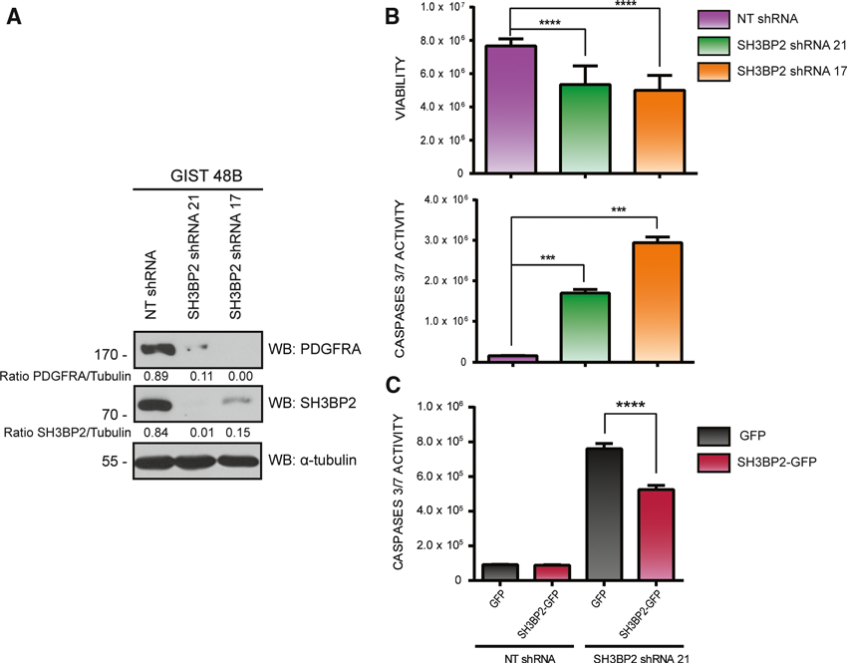
SH3BP2 silencing decreases PDGFRA expression and cell survival in GIST 48B cells. (A) GIST48B cells were transduced with either control NT (Nontarget) shRNA or two different sequences of SH3BP2 shRNA. Samples were analyzed for SH3BP2 and PDGFRA protein levels. Tubulin was used as loading control. (B) Viability and caspase 3/7 activity analysis was performed in NT and SH3BP2 GIST48B‐silenced cells. Statistical significance (***P <* *0.001, ****P <* *0.0001; one‐way ANOVA with Bonferroni's post hoc test; *n *=* *3; mean ± SEM) is relative to NT shRNA. (C) NT and SH3BP2 GIST 48B‐silenced cells were posteriorly transduced with GFP or SH3BP2‐GFP, and caspase 3/7 activity was evaluated. Statistical significance (****P <* *0.0001; unpaired *t*‐test; *n *=* *3; mean ±SEM) is relative to GFP‐transduced cells in each case.

We have shown that SH3BP2 downregulation regulates the viability of GIST cells with activating KIT mutations that are sensitive (GIST882) and resistant (GIST48) to imatinib treatment. To investigate the effects on KIT‐negative GIST cells, we used a GIST48‐derivative line (GIST48B) with undetectable KIT expression due to continuous exposure to the HSP90 inhibitor 17‐AAG (Muhlenberg *et al*., 2009). Our data show that SH3BP2 silencing downregulates PDGFRA expression in GIST48B cells (Fig. 4A) in a similar fashion as in GIST882 cells. In this case, SH3BP2 knockdown also affects cell viability and increases cell apoptosis measured by caspase 3/7 activity (Fig. 4B). Consequently, SH3BP2 reconstitution significantly reduces the apoptosis resulting from SH3BP2 knockdown (Fig. 4C).

### 3.7 Cell migration is impaired after SH3BP2 silencing in GIST cells

**Figure 5:**
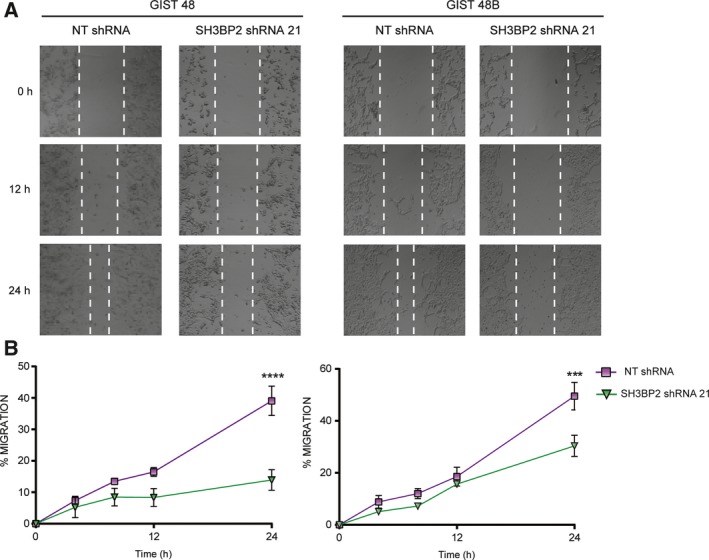
Silencing of SH3BP2 impairs cell motility/migration of GIST cells. (A) Wound‐healing assay was used to assess the effect on cell migration after SH3BP2 silencing in GIST48 and GIST48B cells. The pictures show a representative experiment for each condition at different points. (B) Quantification of migrated cells is represented at different points. Statistical significance (***P <* *0.001, ****P <* *0.0001; unpaired *t*‐test; *n *=* *3; mean ± SEM) is relative to NT shRNA.

A role for SH3BP2 has been reported in cell migration in neutrophils, through Vav and Rac2 GTPase activity (Chen *et al*., 2012). Therefore, we next evaluated the effect of SH3BP2 silencing on the ability of cells to migrate performing a wound‐healing assay. GIST48B cells, although they do not express KIT, also migrate similar to GIST48. Our data show that SH3BP2‐silenced GIST48 and GIST48B cells exhibit a significant decrease in migration, after 24 h of culture (Fig. 5). Altogether, our results indicate that SH3BP2 is important for both survival and motility in GIST cells. We also intended to perform the same approach with GIST882 cells; however, the proliferation rate of these cells was higher than their migration rate, so it was not possible to evaluate the effect of SH3BP2 on GIST882 migration (data not shown).

### 3.8 SH3BP2 silencing prevents tumor growth *in vivo*

**Figure 6:**
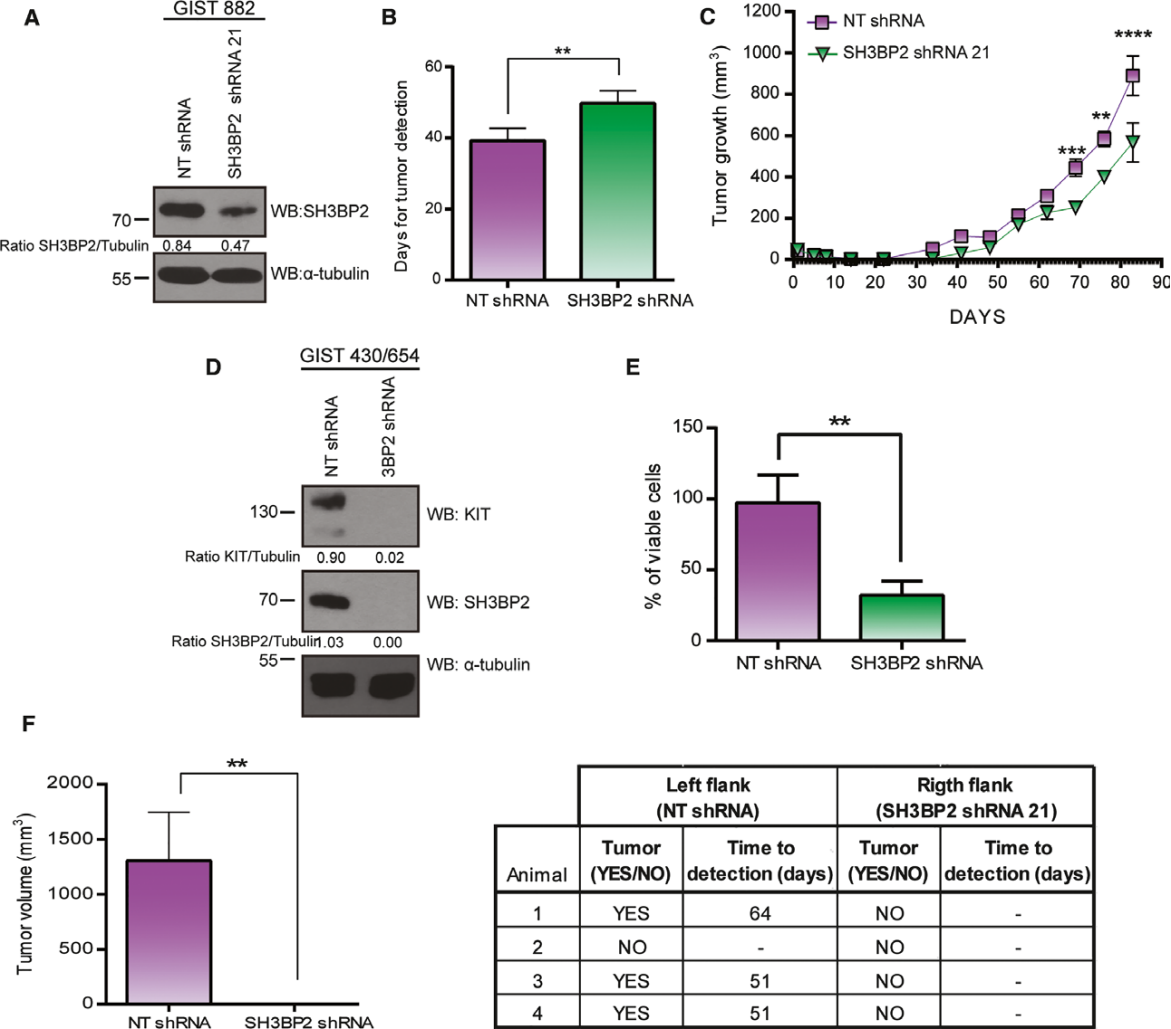
Silencing of SH3BP2 causes a reduction in GIST tumor growth. (A) GIST882 cells were transduced with either control NT (nontarget) shRNA or SH3BP2 shRNA, and the efficiency of SH3BP2 silencing was assessed by western blot the day of the injection. Tubulin was used as loading control. (B) Bars show the day of detection of tumor in NT or SH3BP2‐silenced cells. Statistical significance (**P <* *0.01; unpaired *t*‐test; *n *=* *4 each experimental group; mean ± SEM) is relative to NT shRNA. (C) Diagram shows the rate of tumor growth of NT and SH3BP2‐silenced xenografts. Statistical significance (**P <* *0.01, ***P <* *0.001, ****P <* *0.0001; one‐way ANOVA with Bonferroni's post hoc test; *n *=* *4 each experimental group; mean ± SEM) is relative to NT shRNA at each time point. (D) GIST430/654 cells transduced with either control NT (nontarget) shRNA or SH3BP2 shRNA, and the efficiency of SH3BP2 silencing was evaluated by western blot the day of the injection. Tubulin was used as loading control. (E) Bars represent the percentage of viable cells after 7 days of SH3BP2 silencing *in vitro*. Statistical significance (**P <* *0.01; unpaired *t*‐test; *n *=* *3; mean ± SEM) is relative to NT shRNA. (F) Bars represent tumor volume of NT and SH3BP2‐silenced xenografts at day 80 postinjection, and table shows tumor development in the different animals. Statistical significance (**P <* *0.01; unpaired *t*‐test; *n *=* *3 each experimental group; mean ± SEM) is relative to NT shRNA.

We next tested the ability of SH3BP2 silencing to prevent GIST tumor growth in a xenograft model. To do so, GIST882 cells were transduced with NT shRNA or SH3BP2 shRNA and, after puromycin selection for two days, cells were subcutaneously injected in both flanks of athymic nude mice. NT shRNA control cells were injected in the left flank and SH3BP2 shRNA cells in the right flank. SH3BP2 protein levels were evaluated at the time of the injection (Fig. 6A). Consistent with the results obtained *in vitro*, a significant delay in tumor growth (Fig. 6B) and tumor volume (Fig. 6C) was observed for the SH3BP2 shRNA cells compared to the NT shRNA control cells.

We also analyzed the *in vivo* effect of SH3BP2 silencing in the imatinib‐resistant GIST48 cell line. To do so, we injected control NT and SH3BP2 shRNA‐transduced GIST48 cells in mice as described above for the GIST882 cells. However, these cells failed to form subcutaneous tumors under these conditions. After three months, no tumor growth was observed in any condition. We then evaluated the expression of SH3BP2 and MITF in other GIST cell lines, including imatinib‐sensitive GIST‐T1 and different imatinib‐resistant sublines derived from GIST‐T1 and GIST430/654 cells. Figure S6 shows that SH3BP2 and MITF molecules are expressed in all the GIST cell lines tested.

The imatinib‐resistant GIST cell line GIST430/654 (*KIT* exon 11 delV560‐L576) with a secondary KIT mutation (*KIT* exon 13 V654) and similar kinetics to GIST882 to induce tumors (Smyth *et al*., 2012) was used as an imatinib‐resistant model for tumor growth *in vivo*. Silencing of SH3BP2 on GIST430/654 shows a reduction in KIT expression (Fig. 6D) and a concomitant reduction of cell viability (Fig. 6E) in accordance with the previous results. NT shRNA cells and SH3BP2 shRNA‐silenced cells were injected as described above in BALB/c severe combined immunodeficient (SCID) mice. Interestingly, SH3BP2 shRNA‐silenced cells did not grow in any of the animals (Fig. 6F). These *in vivo* results support the critical role of SH3BP2 in cell survival in an imatinib‐resistant GIST cell line harboring different KIT mutations.

## 4 Discussion

Although GISTs can be successfully treated with imatinib mesylate or other TKIs, new therapeutic options are needed because complete responses are rare and most patients develop resistance to these drugs over time (Poveda *et al*., 2017). Based on our previous finding that the adaptor SH3BP2 was able to regulate KIT and MITF expression and viability on mast cells (Ainsua‐Enrich *et al*., 2015), we explored the ability of this protein to regulate these events in GIST. To date, the expression and functional role of SH3BP2 and MITF in GIST was not known. In this work, we characterize that SH3BP2, and the transcription factor MITF, were expressed in primary GIST tumors and in different GIST cell lines harboring different clinically representative primary and secondary mutations. Interestingly, SH3BP2 was not expressed in the sample from a primary GIST WT; however, as GISTs of this kind are very rare tumors and we were only able to analyze a single sample, we cannot conclude that this result is common to other GIST WT and further experiments would be needed.

The silencing of SH3BP2 had a proapoptotic effect on imatinib‐sensitive GIST882 and imatinib‐resistant GIST48 cell lines, which was concomitant to the downregulation of KIT oncogenic protein levels. Interestingly, SH3BP2 silencing also led to a PDGFRA receptor reduction, indicating that SH3BP2 may additionally modulate both tyrosine kinase receptors and may also be a target for KIT‐negative GISTs. Supporting this notion is the fact that SH3BP2 colocalizes with both receptors in GIST cells. In this context, SH3BP2 may act as a common adaptor in both receptor signaling pathways. We searched for a direct interaction of SH3BP2 and KIT using three hybrid system technologies described elsewhere (Sayos *et al*., 2001), but we were unable to see any direct association (Ainsua‐Enrich *et al*., 2015). Thus, the binding of SH3BP2 to the receptors could be mediated by a common protein partner of these molecules such as Grb2, PLCγ, or PI3K.

Loss of the KIT and PDGFRA oncoproteins has a strong apoptotic effect on GIST cells, as is observed after knocking down these receptors with siRNA or treatment with HSP90 inhibitors (Bauer *et al*., 2006; Liu *et al*., 2007). It is therefore likely that the downregulation of KIT/PDGFRA makes a substantial contribution to the effect of SH3BP2 silencing in GIST cells.

Microphthalmia‐associated transcription factor has been reported to regulate KIT expression and is critical for mast cells differentiation (Tsujimura *et al*., 1996) (Qi *et al*., 2013). It has also been described as an oncogene in melanoma (Garraway *et al*., 2005) and now, for first time, we show its expression in GISTs and its ability to regulate tumor cell viability. The role of MITF in the regulation of PDGFRA transcription has not been previously described, but using patch software, which uses the positional weight matrices from TRANSFAC database (Wingender *et al*., 2000), we found three CATGTG sites—putative MITF binding sites—in the *PDGFRA* promoter at position −1840, −1464, and −1307 (with regard to the TSS described in (Kawagishi *et al*., 1995)). Therefore, it may be the case that MITF modulates PDGFRA transcription, but this possibility needs to be further examined. It has been reported that MITF regulates proliferation and apoptosis through CDK2 and BCL2 in melanoma cells (Du *et al*., 2004; McGill *et al*., 2002). It is conceivable that MITF downregulation by SH3BP2 silencing may also affect GIST cell survival; interestingly, MITF reconstitution in SH3BP2‐silenced cells significantly restored cell survival. Our results identify SH3BP2 as an important mediator of cell survival and a regulator of KIT expression at protein and mRNA level through the control of MITF expression in GIST cells. However, we cannot rule out the possibility that other factors may be involved in SH3BP2‐dependent KIT expression.

It has been shown that the proteasome inhibitor bortezomib causes a transcriptional downregulation of KIT (Bauer *et al*., 2010) and MITF expression in mast cells (Lee *et al*., 2011). Interestingly, we found that bortezomib also induces SH3BP2 and MITF downregulation in GIST882 cells. As bortezomib leads to a wide‐ranging effects on cellular function beyond KIT transcription (Bauer *et al*., 2010), the interpretation of these data, suggesting common regulatory pathways of these proteins, requires caution. Agreeing with the existence of common and reciprocal pathways, it has been reported that there is an inverse regulation between MITF and KIT, through selective miRNA expression. MiR‐539 and miR‐381 have been shown to be down‐regulated by KIT signaling, and they repressed MITF expression through conserved miRNA binding sites in the MITF 3′‐untranslated region in mast cells (Lee *et al*., 2011). KIT signaling through PI3K and PLC gamma is attenuated after SH3BP2 silencing in GIST, so it is conceivable that selective miRNA expression dependent on KIT signaling will be altered as well. Further experiment will be needed to explore miRNA signature in SH3BP2‐silencing context. As the mRNA levels of MITF are not decreased by SH3BP2 silencing, it is likely that SH3BP2 may also act by regulating miRNA, thus affecting the expression of MITF or other molecules involved in KIT/PDGFRA‐dependent events. The fact that MITF mRNA levels are significantly higher in GIST48 after SH3BP2 silencing may be attributed to a compensatory cell mechanism. On the other hand, the transcription factor ETV1 has been reported to be required for GIST cell growth and development of an aggressive phenotype (Chi *et al*., 2010), and its expression correlates with KIT expression (Zhang *et al*., 2014). Indeed, preliminary data from our group show that SH3BP2 silencing reduces ETV1 as well. Interestingly, imatinib treatment reduces SH3BP2 and MITF expression suggesting a positive feed‐forward loop that would support the critical oncogenic signaling pathway in these cells. The axis KIT‐SH3BP2‐MITF was shown to be key for mast cell survival (Ainsua‐Enrich *et al*., 2015).

Our results show that SH3BP2 silencing affects not only cell survival but also cell motility. Interestingly, GIST48B cells, which lack KIT expression, migrate in a similar way to GIST48 cells. As we have shown, GIST48B cells upregulate PDGFRA expression in comparison with GIST48 cells, and this phenomenon may compensate for the lack of KIT; accordingly, PDGFRA appears phosphorylated and presumably activated. SH3BP2 silencing leads to a decreased PDGFRA expression in GIST48B and to a subsequent reduction in cell migration and an increase in cell apoptosis.

Finally, we analyzed the ability of SH3BP2 to inhibit tumor growth *in vivo*. We first performed xenograft experiments with SH3BP2‐silenced GIST882 cells which showed a significant delay in GIST subcutaneous tumor grafting and a reduction in final tumor volume compared to nonsilenced cells. After unsuccessful attempts to perform GIST48‐xenograft models, we used GIST430/654 as an imatinib‐resistant GIST model. Using this cell line, the deleterious effect of SH3BP2 silencing on tumor formation was more evident than in the GIST882 model, probably due to more efficient SH3BP2 silencing. However, in both cases, our results showed a significant impairment in the ability of SH3BP2‐silenced cells to form tumors.

Because most imatinib‐resistant GISTs develop secondary mutations within the KIT or PDGFRA kinase domains, novel therapeutic approaches that do not directly target these kinases are particularly important. The example of HSP90 inhibitors, or bortezomib, which both lead to a loss of KIT oncoprotein expression in GISTs, supports this notion (Bauer *et al*., 2006, 2010). In this respect, we have shown that SH3BP2 regulates KIT and PDGFRA expression, induces apoptosis, and impairs migration in GIST cells. Our results provide compelling information on SH3BP2 as a new selective target against GIST cells harboring various resistance mutations. The use of KIT/PDGFRA‐targeted nanoparticles that encapsulate SH3BP2 siRNA, alone or in combination with tyrosine kinase inhibitors, may constitute a novel therapeutic tool for the treatment of imatinib‐resistant GISTs. Moreover, the use of MITF inhibitors could be useful against GIST development and progression. Finally, both SH3BP2 and MITF may also be useful targets, in other malignancies in which KIT is the driven molecule, such as acute myeloid leukemia (AML), mastocytosis, or certain cases of melanoma.

## 5 Conclusion

Our data show that SH3BP2 is a key molecule involved in the modulation of KIT/PDGFRA expression, cell migration, and viability, both *in vitro* and *in vivo* in GIST. This suggests that SH3BP2 or SH3BP2 partners may be potential targets for GIST treatment and other malignancies in which KIT/PDGFRA are mutated.

## Author contributions

ESC, EAE, and MM conceived the experiments and wrote the manuscript. ESC, EAE, ANF, PR, and AGV performed the experiments. SB and IM provided technical support. JA, DA, JS, and CS provided reagents and technical support and MM secured funding. All authors reviewed the manuscript.

## Supporting information


**Fig. S1.** Phosphorylation status of KIT and PDGFRA in GIST882, GIST48 and GIST48B cells.
**Fig. S2.** SH3BP2 colocalizes with KIT and PDGFRA in GIST cells.
**Fig. S3.** PLC gamma and AKT phosphorylation are affected in SH3BP2‐silenced GIST882 and GIST48 cells.
**Fig. S4.** Evaluation of reconstitution efficiency of GIST48 SH3BP2 silenced cells.
**Fig. S5.** Imatinib and bortezomib treatment of GIST882 reduces SH3BP2 and MITF levels.
**Fig. S6.** SH3BP2 and MITF are expressed in GIST cell lines with different mutations.
