# Supplementary material for: Silencing of adaptor protein SH3BP2 reduces KIT/PDGFRA receptors expression and impairs gastrointestinal stromal tumors growth
Source: Mol Oncol. 2018 Jun 30;12(8):1383–97. doi: 10.1002/1878-0261.12332 (PMC6068349; doi:10.1002/1878-0261.12332)
Supplement: Supplementary file 1 — Fig. S1. Phosphorylation status of KIT and PDGFRA in GIST882, GIST48 and GIST48B cells. Fig. S2. SH3BP2 colocalizes with KIT and PDGFRA in GIST cells. Fig. S3. PLC gamma and AKT phosphorylation are affected in SH3BP2‐silenced GIST882 and GIST48 cells. Fig. S4. Evaluation of reconstitution efficiency of GIST48 SH3BP2 silenced cells. Fig. S5. Imatinib and bortezomib treatment of GIST882 reduces SH3BP2 and MITF levels. Fig. S6. SH3BP2 and MITF are expressed in GIST cell lines with different mutations. [file MOL2-12-1383-s001.pdf]

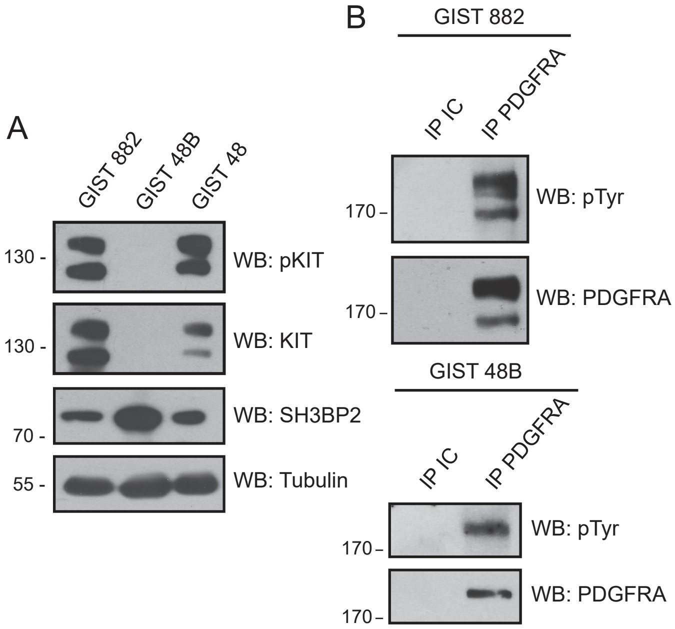

Supplementary Figure 1. Phosphorylation status of KIT and PDGFRA in GIST882, GIST48 and GIST48B cells.

A) Whole cell lysates of GIST882, GIST48 and GIST48B cell lines were analyzed for pKIT, KIT and SH3BP2 expression. Tubulin was used as loading control. B) GIST882 and GIST48B were lysed and immunoprecipitated with anti-PDGFRA or with a rabbit IgG as a control (Isotype control (IC)). Membrane was blotted with indicated antibodies.

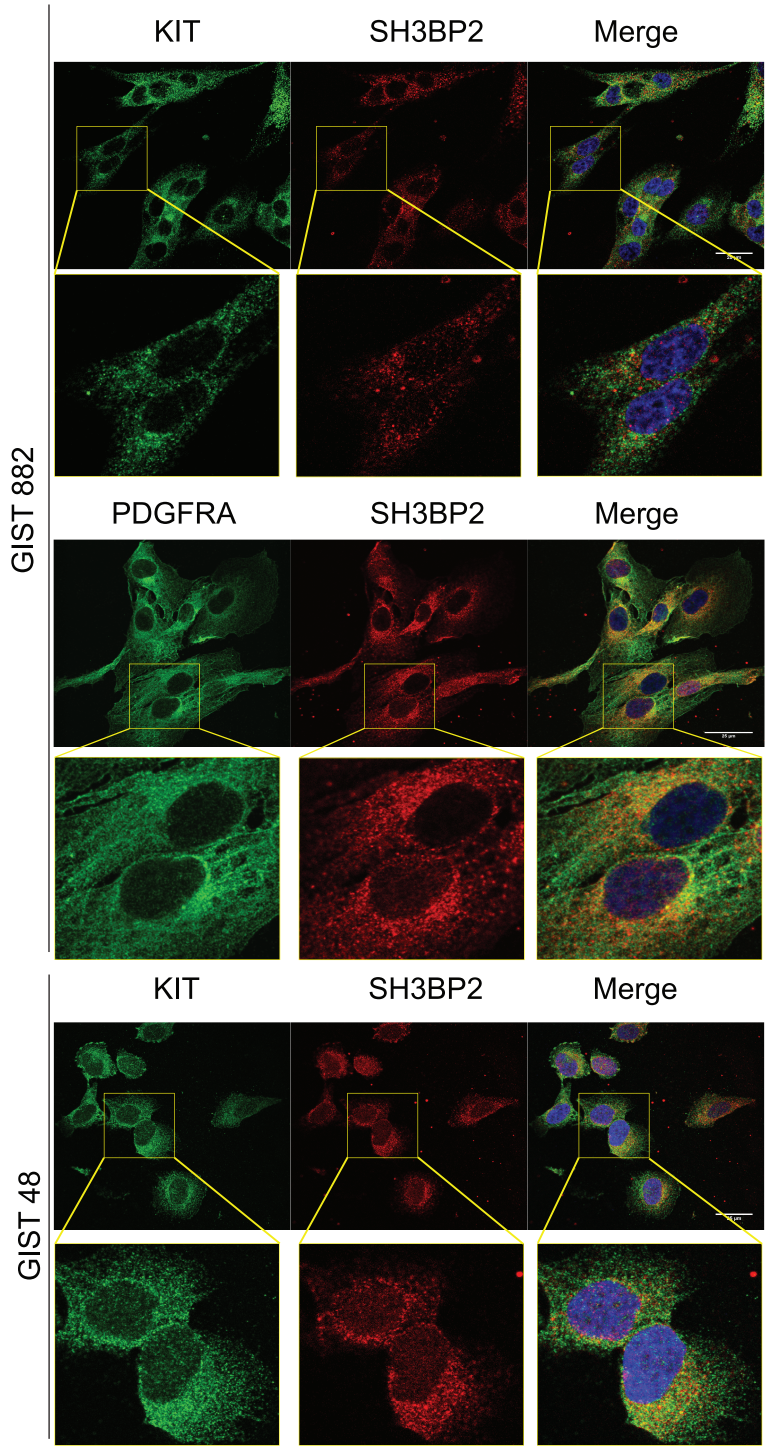

Supplementary Figure 2: SH3BP2 colocalizes with KIT and PDGFRA in GIST cells.
Localization of SH3BP2 (red) and KIT (green) or SH3BP2 (red) and PDGFRA (green) was analyzed by immunofluorescence in GIST882 and GIST48 cell lines. Scale bar, 25 µm.

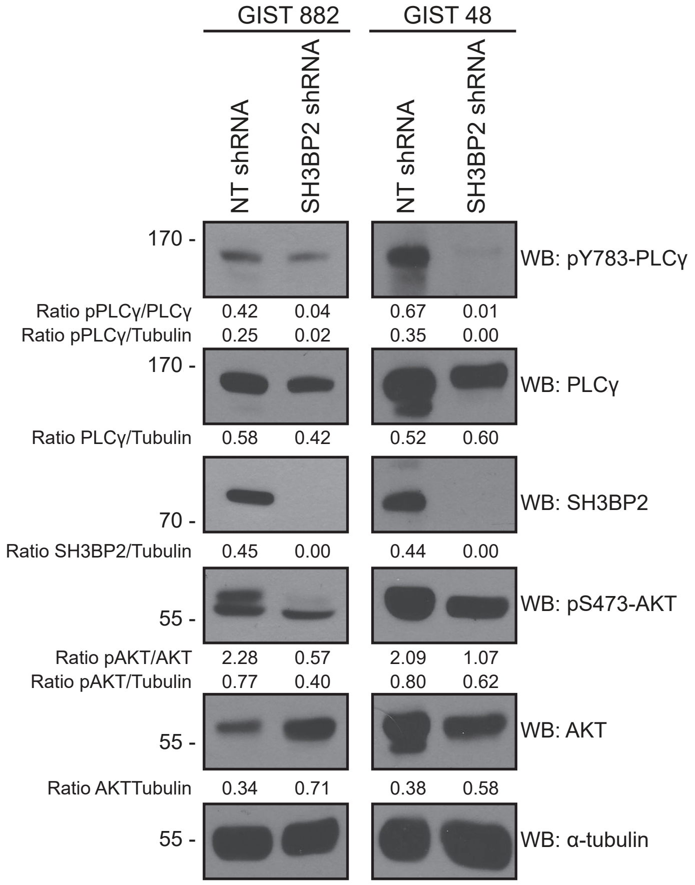

Supplementary Figure 3. PLC gamma and AKT phosphorylation are affected in SH3BP2-silenced GIST882 and GIST48 cells.
GIST882 and GIST48 were transduced with either control NT (Non-target) shRNA or SH3BP2 shRNA. Cell lysates were analyzed for pPLCγY783 and pAKTS473. Total PLCγ, AKT and rpS6, as well as tubulin were used as loading controls.

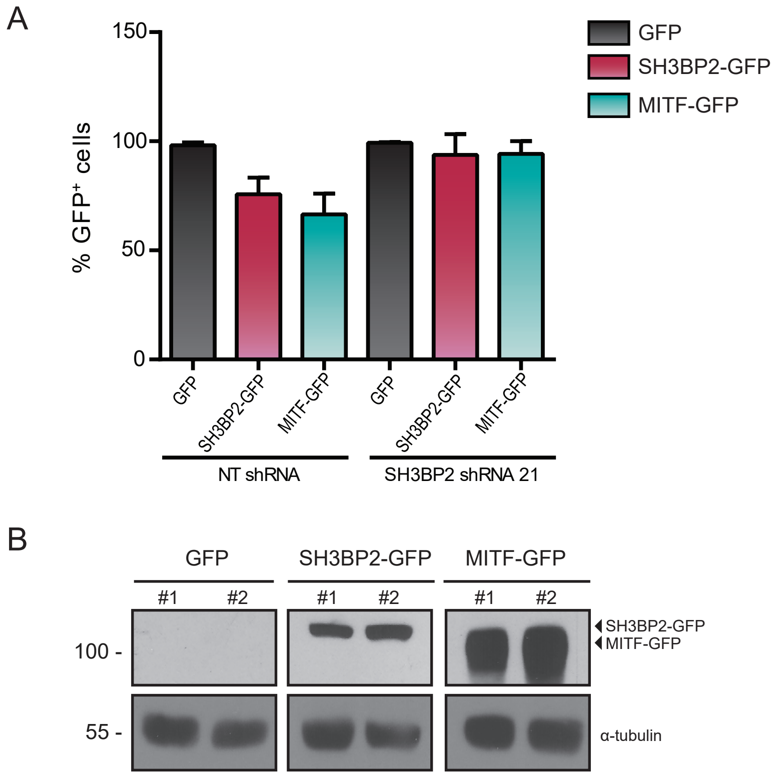

Supplementary Figure 4. Evaluation of reconstitution efficiency of GIST48 SH3BP2 silenced cells.

A) GIST48 cells transduced with either control NT (Non-target) shRNA or SH3BP2 shRNA were posteriorly reconstituted with GFP, SH3BP2-GFP, or MITF-GFP. Percentage of GFP+ cells was evaluated by flow cytometry (n=4 for GFP and SH3BP2-GFP group and n=3 for MITF-GFP group). C) Proper expression of SH3BP2-GFP and MITF-GFP was checked by WB. Tubulin was used as loading control.

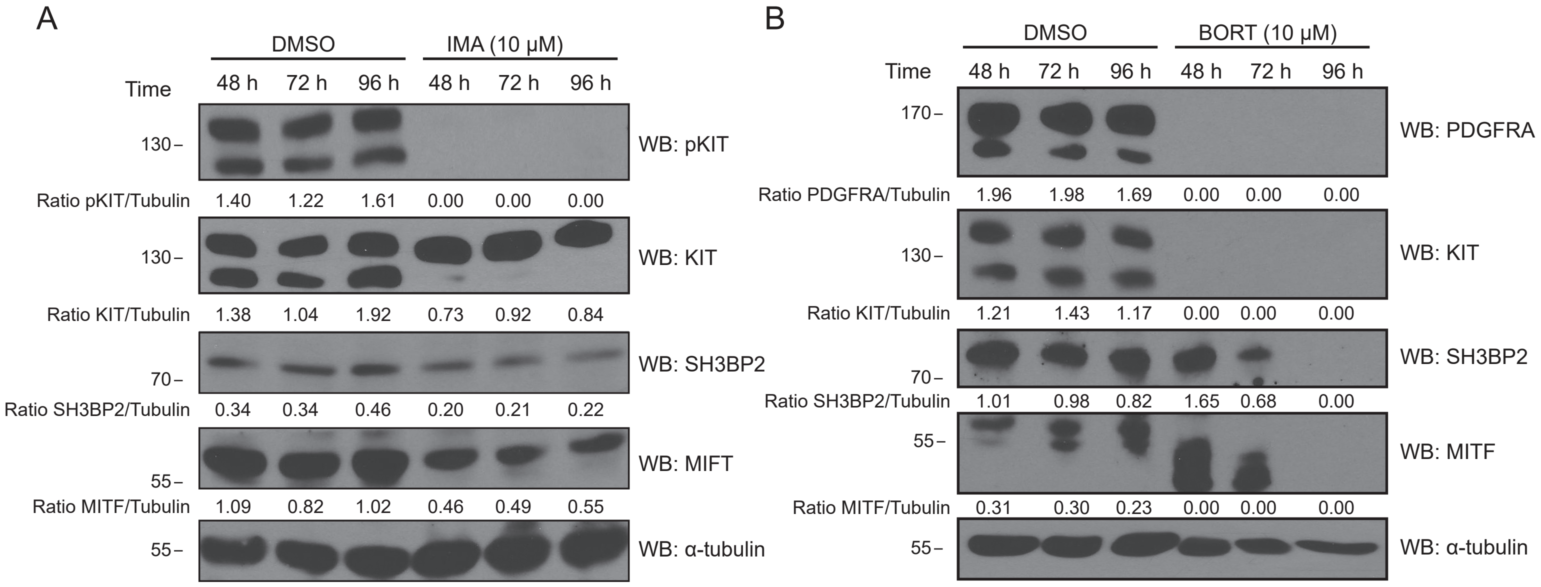

Supplementary Figure 5: Imatinib and bortezomib treatment of GIST882 reduces SH3BP2 and MITF levels.
GIST882 were treated with 10 µM of imatinib (IMA) (A) or bortezomib (BORT) (B) for 48, 72 and 96 h. Protein levels of PDGFRA, pKIT, KIT, SH3BP2 and MITF were analyzed. Tubulin was used as a loading control.

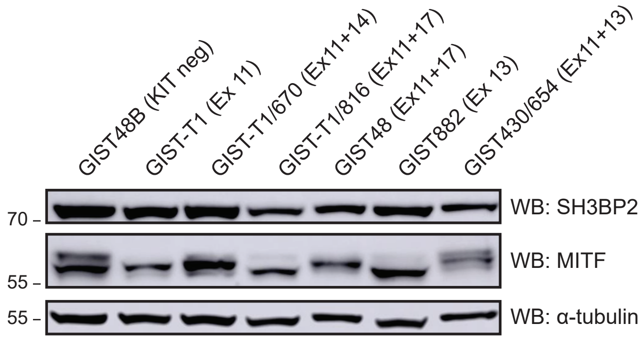

Supplementary Figure 6: SH3BP2 and MITF are expressed in GIST cell lines with different mutations.

Whole cell lysates of different GIST cell lines were analyzed for SH3BP2, and MITF expression. Tubulin was used as loading control.
